# Accelerating Combinatorial Electrocatalyst Discovery with Bayesian Optimization: a Case Study in the Quaternary System Ni‐Pd‐Pt‐Ru for the Oxygen Evolution Reaction

**DOI:** 10.1002/advs.202507302

**Published:** 2025-06-29

**Authors:** Felix Thelen, Rico Zehl, Ridha Zerdoumi, Jan Lukas Bürgel, Lars Banko, Wolfgang Schuhmann, Alfred Ludwig

**Affiliations:** ^1^ Chair for Materials Discovery and Interfaces Institute for Materials Faculty of Mechanical Engineering Ruhr University Bochum Universitätsstraße 150 44801 Bochum Germany; ^2^ Analytical Chemistry– Center for Electrochemical Sciences (CES) Faculty of Chemistry and Biochemistry Ruhr University Bochum Universitätsstraße 150 44801 Bochum Germany

**Keywords:** bayesian optimization, combinatorial materials discovery, compositionally complex solid solutions, electrocatalysis, oxygen evolution reaction

## Abstract

The discovery of high‐performance electrocatalysts is crucial for advancing sustainable energy technologies. Compositionally complex solid solutions comprising multiple metals offer promising catalytic properties, yet their exploration is challenging due to the combinatorial explosion of possible compositions. In this work, human‐decision driven combinatorial sputtering of thin‐film materials libraries and high‐throughput characterization is combined with Bayesian optimization to efficiently explore the quaternary composition space Ni‐Pd‐Pt‐Ru for the oxygen evolution reaction (OER) in alkaline media. Using this method, the global activity optimum of pure Ru is identified after covering less than 20% of the complete composition space with six materials libraries. Six additional libraries are fabricated to validate the activity trend. The resulting dataset is used to formulate general guidelines for the efficient composition of space exploration with combinatorial synthesis paired with Bayesian optimization.

## Graphical abstract

Exploring vast multinary composition spaces for electrocatalyst discovery is experimentally demanding. This study demonstrates how combinatorial synthesis, high‐throughput characterization, and Bayesian optimization can be combined to accelerate materials discovery. With this approach, the global oxygen evolution reaction activity maximum in the Ni‐Pd‐Pt‐Ru system is efficiently identified after the fabrication and characterization of six materials libraries.

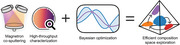

## 1 Introduction

The search for high‐performance electrocatalysts is one of the key challenges in sustainable energy research. Many of the best‐known catalysts rely on scarce and expensive elements, limiting their large‐scale application. Compositionally complex solid solutions, which are quaternary, quinary, or higher‐order systems with multiple elements, frequently present in nearly‐equiatomic ratios, offer a promising pathway to overcoming these limitations by enabling tunable electronic structures and adsorption properties^[^ ^1^ ^]^ However, even when considering a resolution of 5 at.%, a quaternary system already contains 1771 possible compositions, while a quinary system expands to 10626 compositions. Therefore, identifying optimal compositions within the vast composition space of multinary systems is challenging, making traditional experimental approaches impractical.^[^ ^2^ ^]^

**Figure 1:**
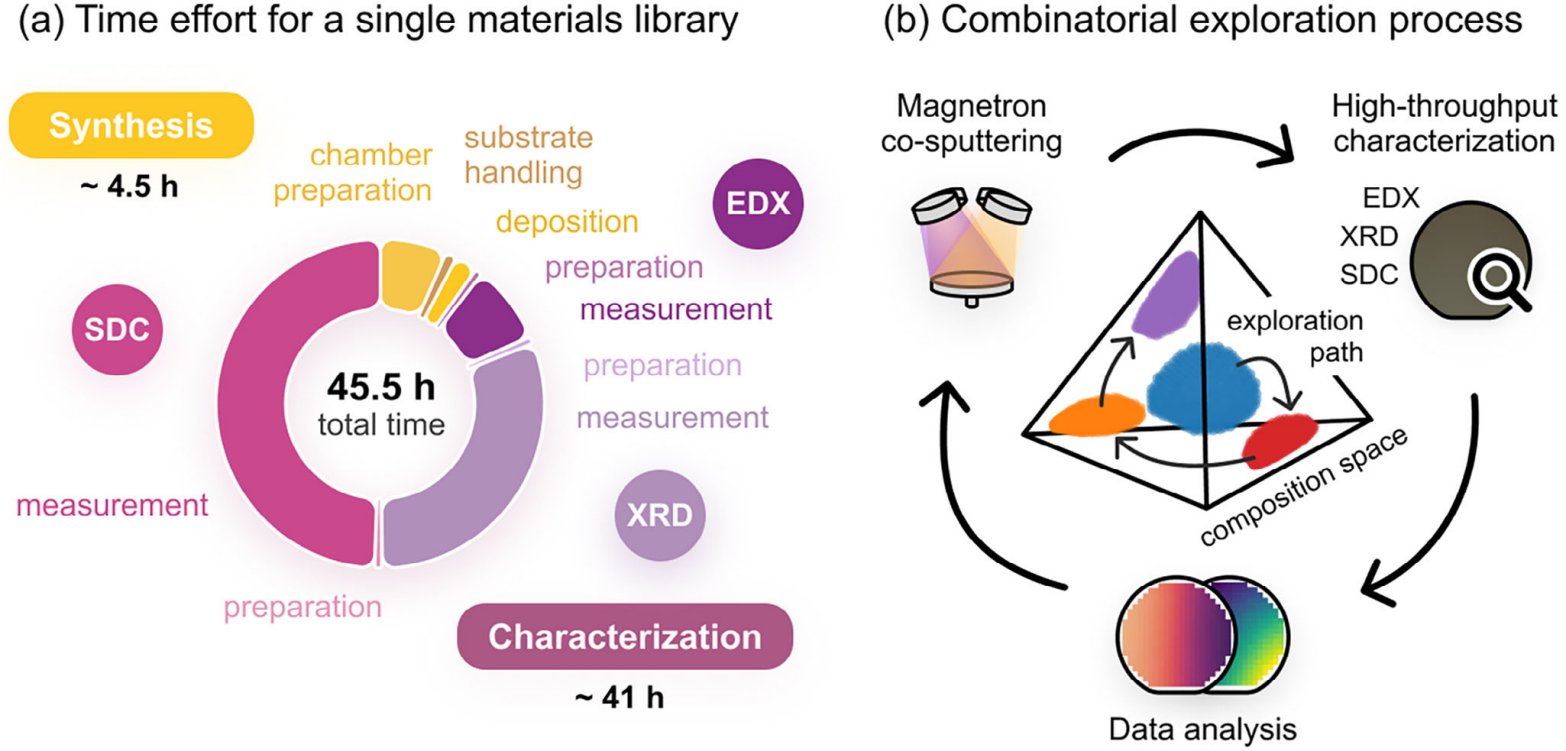
a) Estimated time required for the synthesis and characterization of a single materials library for electrochemical applications. While the deposition is fast, characterization takes significantly longer. b) Schematic of the iterative exploration cycle, where multiple libraries are fabricated to cover the composition space. The next library is fabricated based on the already acquired data.

Combinatorial materials synthesis techniques, such as magnetron co‐sputtering,^[^ ^3^ ^]^ can address this challenge by enabling the fabrication of large numbers of well‐defined compositions in parallel. These methods produce materials libraries, which consist of thin‐film continuous or discrete compositional gradients on a single substrate. Paired with high‐throughput characterization (screening) methods, which allow the automated simultaneous or serial investigation of hundreds of compositions, the combinatorial approach can improve the efficiency significantly compared to one‐at‐a‐time experiments.^[^ ^4^ ^]^ **Figure** **1**a summarizes the time and effort required for the synthesis and characterization of a single materials library for electrochemical applications with 342 pre‐defined measurement areas. While synthesis is relatively fast, characterization is often the most time‐consuming step, even when limited to essential techniques. Energy‐dispersive X‐ray spectroscopy (EDX), used to determine the thin‐film (volume) composition, is one of the shortest characterization steps. X‐ray diffraction (XRD) follows, providing insights into the material's crystal structure and phase constitution. The electrochemical screening, performed using a scanning droplet cell (SDC) setup,^[^ ^5^ ^]^ requires the longest time. The overall process, excluding analysis time, typically spans ≈2 days. While this high‐throughput approach significantly accelerates materials discovery and hundreds of compositions can be fabricated in parallel, it is still constrained by the combinatorial explosion. In quaternary or higher‐order systems, a single materials library can only cover a limited fraction of the composition space: a quaternary library typically spans ≈5%, while the coverage of a quinary system can be less than 1%.^[^ ^6^ ^]^ Consequently, exploring the composition space requires the iterative fabrication of multiple materials libraries, each covering a distinct compositional region. The resulting exploration cycle is summarized in Figure 1b. By varying the cathode powers in each deposition, materials libraries can be fabricated with different compositional ranges: each library corresponds to a hyperbolic paraboloid plane in the multidimensional composition space.

Given these constraints, the fabrication of each new materials library must be guided by the data already acquired from previous experiments. However, navigating multidimensional composition spaces—where both compositional trends as well as electrochemical activity distributions must be considered—is a significant challenge for human intuition alone. Bayesian optimization can be a powerful framework to assist researchers in this task by supporting the exploration cycle of fabrication, characterization, and analysis, complementing human knowledge and/or intuition with data‐driven insights. With this approach, a machine learning model can predict the unknown regions of the composition space based on the previously acquired data in this system.^[^ ^7^ ^]^ The model selects new compositions for fabrication using an acquisition function, such as the expected improvement function. This determines whether the next experiment should focus on exploring less‐characterized regions of the composition space or exploiting areas that already show promising activity.^[^ ^8^, ^9^ ^]^ Mostly, a Gaussian process is used for the underlying machine learning model due to its flexibility and ability of uncertainty quantification independent from the actual observations. Additionally, Gaussian processes are well suited for small and high‐dimensional datasets.^[^ ^8^, ^10^ ^]^

Due to its similarity to the materials exploration cycle, Bayesian optimization has been frequently applied in materials discovery.^[^ ^11^, ^12^, ^13^, ^14^, ^15^, ^16^, ^17^, ^18^, ^19^, ^20^ ^]^ Pairing it with combinatorial synthesis and high‐throughput characterization techniques offers a key advantage: instead of improving the model one composition at a time, entire compositional gradients can be added to the training data in a single iteration. This enhances the efficiency of data collection for materials exploration, accelerating the data‐driven search for new catalysts.

## 2 Results

The combinatorial composition space exploration was performed in the quaternary system Ni‐Pd‐Pt‐Ru to efficiently identify the most electrocatalytically active composition for the OER in alkaline media. The constituents were selected based on a recent exploration of the Ag‐Pd‐Pt‐Ru system.^[^ ^21^ ^]^ By the substitution of Ag with Ni, a more earth‐abundant and cost‐effective alternative element was chosen, while keeping the same elemental face‐centered cubic crystal structure of Ag. The resulting system includes both highly active (Ru) and less active (Pd, Pt) components for the OER, making it a compelling and chemically diverse test framework to evaluate the Bayesian optimization‐driven composition space exploration. The materials libraries were fabricated in a magnetron co‐sputtering system with four cathodes. The chemical compositions of the libraries were characterized by EDX and the electrocatalytic activity with SDC measurements. The Bayesian optimization loop was implemented with a standard Gaussian process and the expected improvement acquisition function. The model was supplied with the measured compositions as input training data. Since the Gaussian process cannot handle voltammetric data—such as linear sweep voltammograms (LSVs) obtained by SDC measurements—out of the box, the current densities at a potential of 1.7 V versus the reversible hydrogen electrode (RHE) were extracted from the LSVs. These values were used as a 1D activity measure and added to the model for the output training data. A detailed description of the experimental methods as well as the Bayesian optimization model can be found in the Supporting Information.

The expected improvement acquisition function allows for balancing the uncertainty of the model and the already found activity maximum. However, during the first few learning iterations, the algorithm prioritizes exploration of the corners of the composition space due to the overall large search space and the resulting high uncertainty of the Gaussian process in these regions. Therefore, initializing the Bayesian optimization with *n* + 1 materials libraries covering the center as well as the corners of the compositional space reduces the experimental time. In order to assess the coverage of the libraries in the composition space, a coverage measure was developed based on a k‐nearest neighbor algorithm. More details are provided in the Supporting Information.

**Figure 2:**
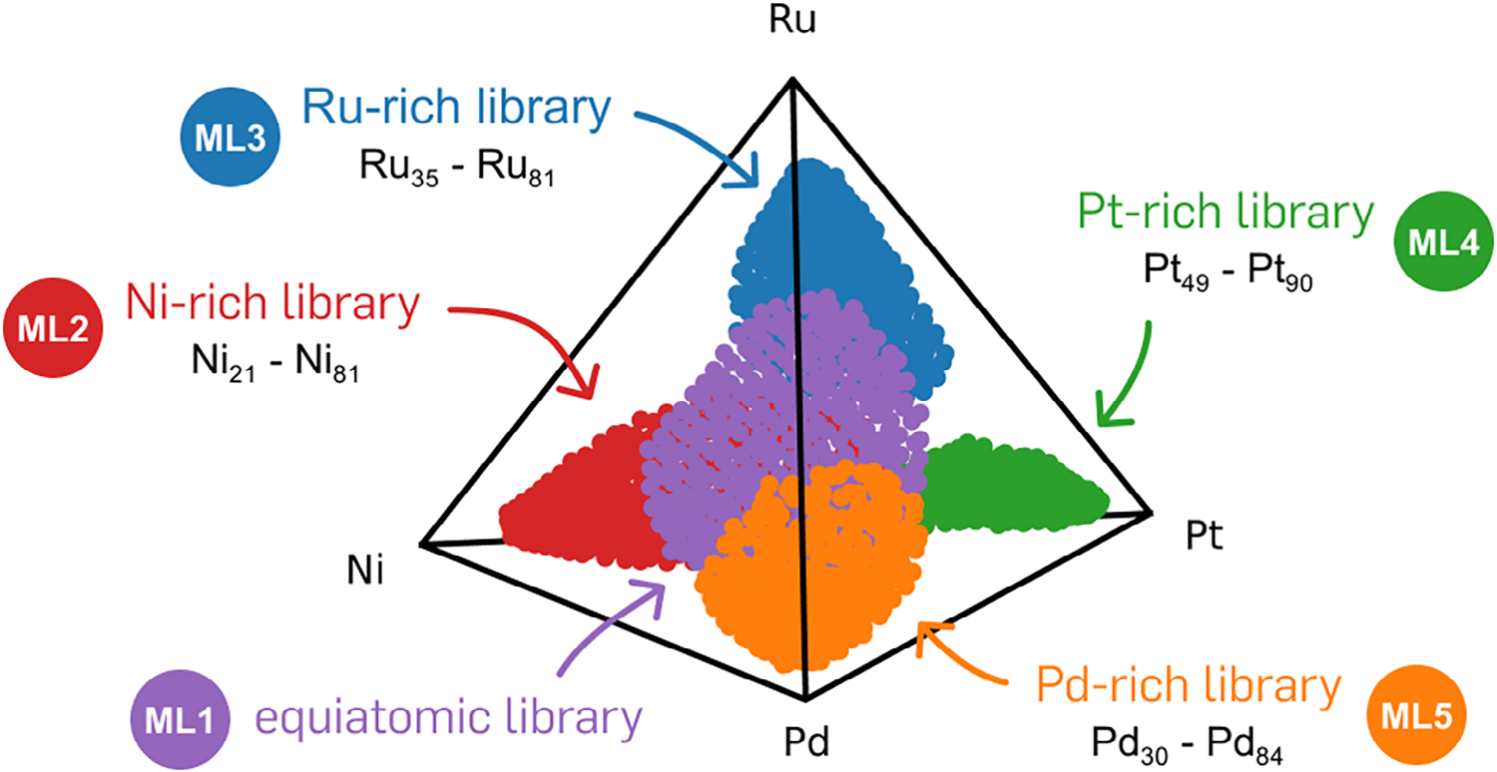
Compositions of the five initial materials libraries plotted in the quaternary composition space. The equiatomic library (purple), the Ru‐rich (blue) as well as the Pd‐rich library (orange) feature overlapping compositions. In this 2D representation of the tetrahedron, the Ni‐rich and Pt‐rich libraries appear to overlap with the others, although this is not the case when viewed in 3D. The subscript values indicate the content of each dominating element in the materials libraries in atomic percent. An animated version of this plot can be found in the Supporting Information.

The compositions of the five initial materials libraries are visualized in the quaternary composition space in **Figure** **2**. The libraries cover 18.6% of the quaternary composition space. The equiatomic library (ML1) achieves the highest compositional coverage with 5.7%, while the Pt‐rich library (ML4) shows the smallest coverage of 2%. Due to the simultaneous operation of all four cathodes during co‐sputtering, compositions at the extreme boundaries of the quaternary space are inaccessible.

**Figure 3:**
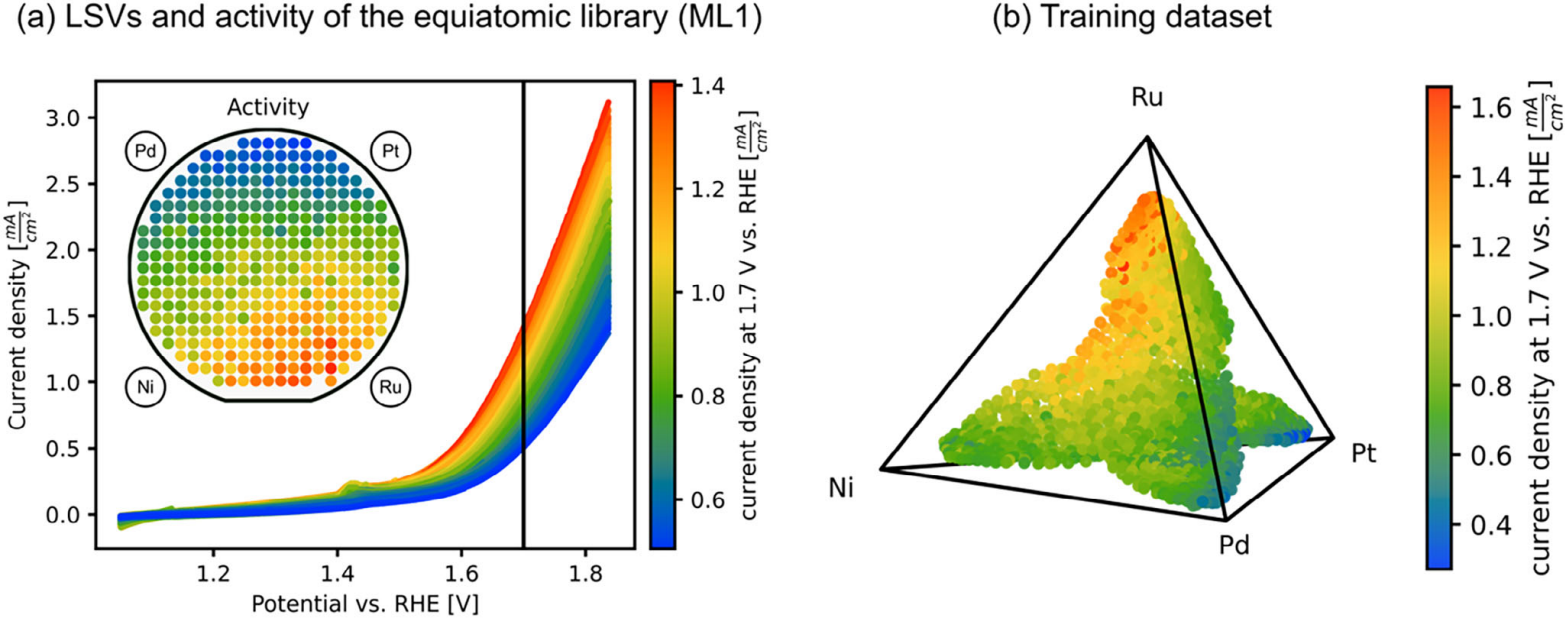
a) LSVs obtained from the SDC measurements of the equiatomic materials library (ML1). The activity is determined by the current density at 1.7 V. The sputter target positions (shown in the inset together with the color‐coded activity plotted for all 342 measurement areas of ML1) in the fabrication process were identical across all depositions. Activity increases with increasing Ru content. By combining the activity with the compositions of all five materials libraries, the training dataset for the Bayesian optimization algorithm, visualized in b), is created. The dataset consists of 1685 samples, where each sample includes a quaternary composition vector as input features and a corresponding electrochemical activity as the target value.

**Figure** **3**a exemplarily shows the LSVs obtained from SDC measurements of the equiatomic materials library, color‐coded by their current density at a potential of 1.7 V versus RHE, and the resulting activity distribution on ML1. The activity in this library increases with higher Ru‐contents. Figure 3b combines compositional data with measured activities, forming the training dataset for the Bayesian optimization algorithm. The highest measured activity of 1.65 mA cm^−2^ was found in the Ru‐rich library within the high Ru content region. Toward the equiatomic library, the activity decreases gradually, forming a smooth activity gradient.

**Figure 4:**
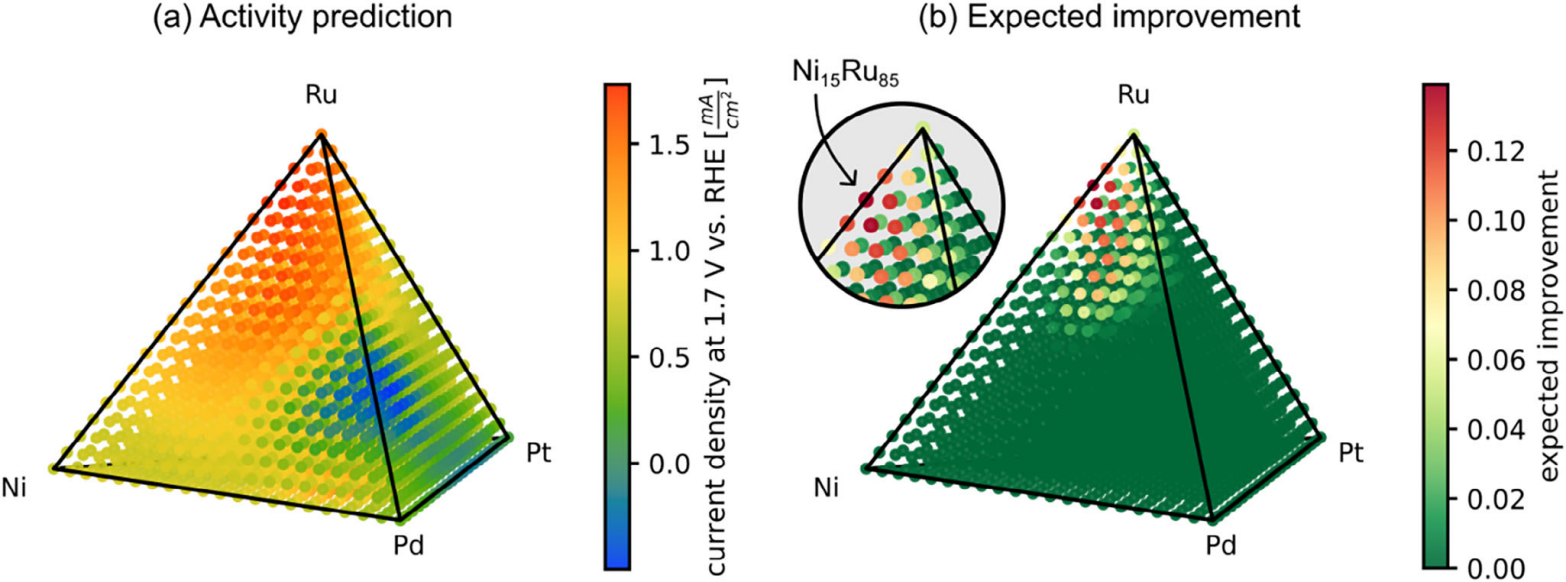
a) Activities predicted by the Gaussian process based on the five initial materials libraries (ML1‐5). The activity trend points toward compositions with a high Ru‐content and with higher contents of Ni than Pd or Pt. b) The expected improvement calculated from the activity prediction and the model's uncertainty in the composition space sampled at 5 at.% steps. The composition with the highest expected improvement is Ni_15_Ru_85_.

After training, the Gaussian process model can predict the activity across the remaining composition space, as illustrated in **Figure** **4**a. The predictions are performed on a test dataset with 1771 compositions evenly distributed in the quaternary composition space with 5 at.% steps in between each composition. Due to the model's unconstrained output space, negative current densities are occasionally predicted, notably in the compositional region near Pd₂Ru. Consistent with the training dataset, the model predicts high activity for compositions with higher Ru content. While the incorporation of Pd and Pt shows no positive impact on activity, the model suggests that Ni contents ranging from 5 to 20 at.% may enhance performance. Since the model also smooths the training data, it effectively removes experimental noise, which can result in predicted activity values lower than the highest measured activity. Together with the uncertainty of the model, the prediction is used to compute the expected improvement acquisition function on the composition space, indicating in which compositional range to sample next. This is visualized in Figure 4b. A high expected improvement was found in the ternary Ni‐Pd‐Ru region with high Ru content. The composition showing the highest expected improvement is Ni_15_Ru_85_.

**Figure 5:**
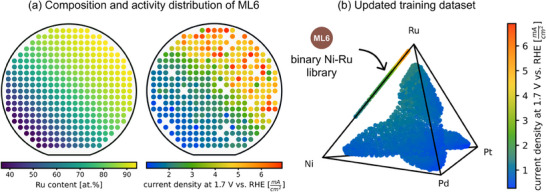
a) Compositional distribution and electrochemical activity of the Ni‐Ru materials library (ML6) plotted for all 342 measurement areas (color code for Ru content and electrochemical activity at a fixed potential) and b) the updated activity map for the quaternary composition space showing the new data points at the edge of the tetrahedron. (b) displays the same dataset as Figure 3b, but with the addition of ML6, which strongly dominates the color scale due to its significantly higher activity. 322 compositions were added to the initial dataset, which covers 19.4% of the composition space. ML6 suggests a global activity maximum toward pure Ru.

Guided by the model's prediction, we selected the binary Ni‐Ru system for the next materials library. **Figure** **5**a shows the compositional distribution and corresponding electrochemical activity of ML6. The binary composition spread exhibits a gradient with high Ru content in the top right and high Ni content in the bottom left of the library, resulting in an activity distribution symmetric along the diagonal. As expected, higher Ru contents correlate with increased activity. Figure 5b illustrates the updated composition space, where the newly acquired data points are positioned at one of the edges of the tetrahedron. The activities measured in ML6 surpass those of all previously tested compositions, and the observed trend indicates a global activity maximum at pure Ru. The binary library extends the total experimental coverage of the composition space by 0.8% to 19.4%.

**Figure 6:**
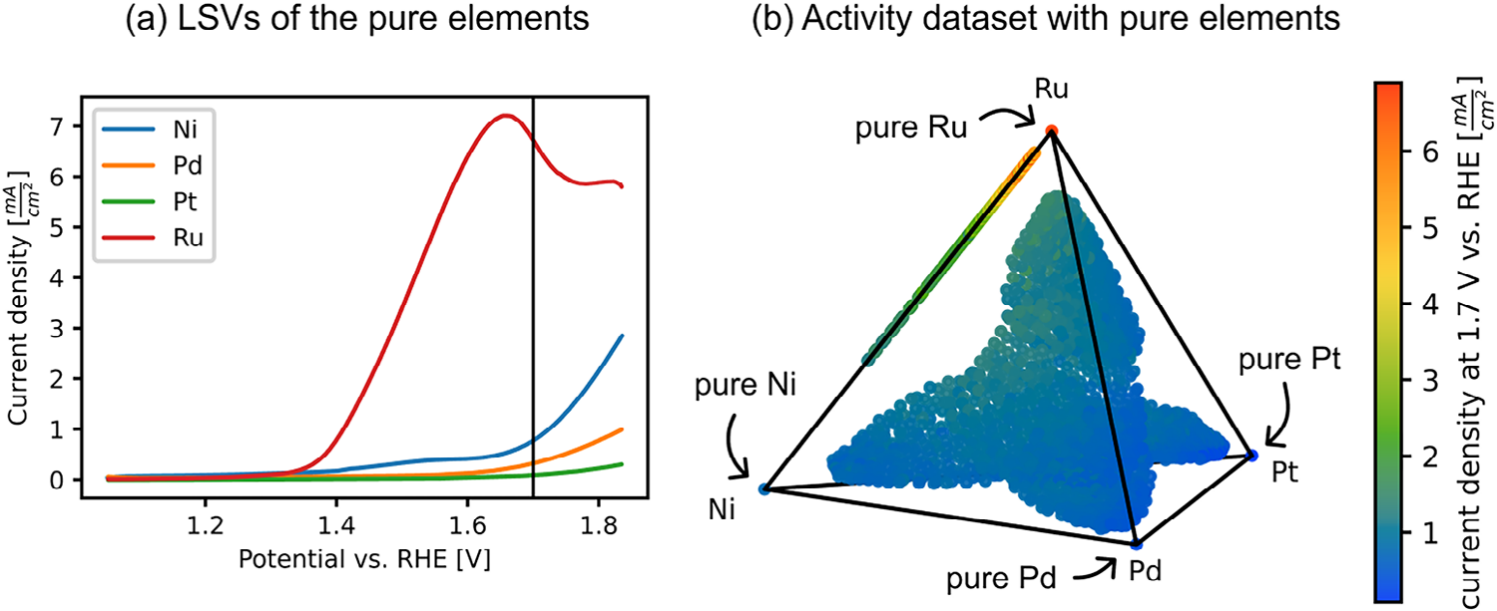
Measured LSVs of the pure elements a) and updated activity distribution in the composition space (b). The vertical line in (a) highlights the potential of 1.7 V versus RHE, which is plotted in (b). Pure Ru is the best catalyst in the system.

In order to verify the activity maximum of pure Ru as the result of the search in the quaternary space, the activities of all four pure elements were determined. For each element, a thin film was prepared in the same sputter chamber using the same process temperature and pressure as for the materials libraries. Three individual SDC measurements were performed and the resulting LSVs were averaged. **Figure** **6** shows the LSVs and the updated activity dataset. Among the four elements, pure Ru exhibits the highest activity characterized by a rapid increase in current density, peaking just below 1.7 V versus RHE. Ni shows moderate activity, with a less steep yet noticeable rise in current density at higher potentials, while Pd and Pt exhibit the lowest OER activity. The plateau observed in the LSV of Ru suggests that mass transport limitations or gas bubble formation may affect performance at elevated potentials. Incorporating the activities of the pure elements into the dataset, as illustrated in Figure 6b in the corners of the tetrahedron, reveals that the observations align well with the overall activity trend, with pure Ru being the best‐performing catalyst in the Ni‐Pd‐Pt‐Ru system.

**Figure 7:**
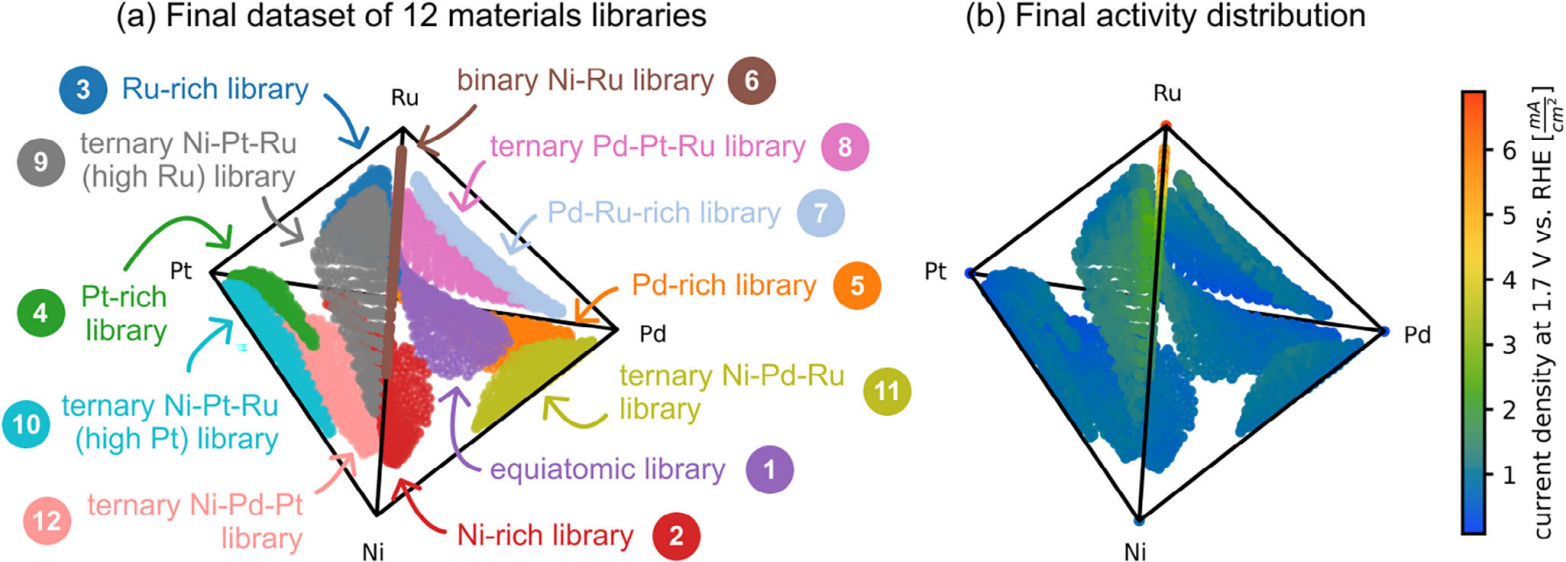
a) Final dataset of 12 materials libraries covering 36.7% of the composition space. The final dataset consists of 4030 compositions. b) Activity distribution in the composition space. Compared to other shown composition space plots, these figures are rotated to make all libraries visible.

To validate the global activity maximum of pure Ru in the Ni‐Pd‐Pt‐Ru space, six additional materials libraries (ML7‐12) were fabricated in regions with large distances from the already acquired compositions. **Figure** **7** shows the fabricated compositions alongside the activity distribution in the composition space. In total, more than 4000 compositions were acquired from the 12 materials libraries, covering 36.7% of the quaternary composition space. Five libraries were fabricated in all four ternary subspaces (ML8‐12), while ML7 covers the compositional area around the highest uncertainty of the Gaussian process after the last training iteration. The additional libraries confirm the already found global activity maximum of pure Ru.

## 3 Discussion

By combining combinatorial synthesis and high‐throughput characterization with Bayesian optimization, the global activity maximum in the Ni‐Pd‐Pt‐Ru composition space was successfully identified after only six materials libraries. Remarkably, exploration of only 20% of the composition space is needed to identify the optimal composition. This demonstrates that despite the vast size of multinary composition spaces, efficient exploration can significantly reduce experimental effort. Given that the fabrication and characterization of a single materials library requires ≈2 days, the total time investment for this exploration can be estimated to be 12 days. The six additional libraries allowed for a systematic validation, confirming pure Ru as the global activity maximum in the system. This finding aligns with Ru's well‐established role as one of the most efficient OER catalysts. RuO_2_, which forms under reaction conditions, is located close to the top of the volcano plot^[^ ^22^ ^]^ and therefore features nearly optimal binding energies for the reaction intermediates. The incorporation of Ni, Pd, and Pt to form compositionally complex solid solutions could have led to a better‐performing catalyst. However, in the specific system that we investigated here, compositional complexity did not enhance catalytic activity compared to the best‐performing constituent element Ru. Instead, the additional elements likely disrupted the nearly optimal binding energy characteristic for Ru, leading to the observed activity trend in the quaternary system Ni‐Pd‐Pt‐Ru. Additionally, the statistical replacement of active Ru centers through the incorporation of less active elements could have also contributed to the reduction of the overall activity.

**Figure 8:**
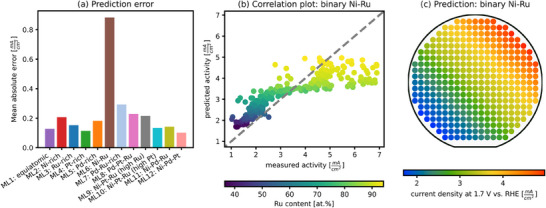
Evaluation of the model's extrapolation performance using a leave‐one‐library‐out approach. For each iteration, one materials library was excluded from the training dataset, and the model was trained on the remaining libraries. a) Mean absolute error (MAE) of the predictions for each excluded library. b) Parity plot showing predicted versus measured activity for the excluded Ni‐Ru library (ML6), none of these points were part of the training set. This library showed the highest prediction error among all. c) Predicted activity distribution across the Ni‐Ru composition space for the same test case.

Although the global activity maximum was identified with a relatively small experimental effort, the twelve materials libraries are likely insufficient to accurately predict the remaining composition space. This limitation becomes evident when performing a train‐test split, where the model is trained on eleven libraries and tasked with predicting the excluded one. **Figure** **8**a shows the prediction error of each excluded materials library. While the model captures the activity trend for each library, the absolute predicted values deviate significantly, resulting in a relatively high mean absolute error. This is particularly notable as the median activity range of the libraries is 1.2 mA cm^−2^. The Ni‐Ru library (ML6) exhibits the highest prediction error by a wide margin. A comparison of Figure 8c and Figure 5a reveals that while the model correctly predicts the trend of increasing activity with higher Ru content for this library, lower activity values are consistently overestimated, whereas higher activities are significantly underestimated.

Several factors contribute to this discrepancy. One major limitation could be missing features in the training data to successfully predict the catalytic activity. As the activity is predominantly a surface‐related property, compositions acquired by EDX only serve as a proxy for the surface composition in the predictions. However, the surface composition can differ from the volume of the film due to surface segregation. While X‐ray photoelectron spectroscopy (XPS) could provide insights into the surface composition, its measurement and analysis time remains significantly longer than other high‐throughput techniques like EDX, making it impractical for measuring entire materials libraries. A recent study demonstrates that surface composition can instead be predicted from volume composition using Gaussian process regression, substantially reducing the effort needed to characterize the surface of entire materials libraries. Additionally, surface morphology and crystal structure also influence catalytic performance. The XRD analysis reveals that most of the composition space shows an fcc crystal structure. However, in the Ru‐rich regions of ML7 and ML9, particularly in the Ru‐rich library (ML3) and the binary Ni‐Ru library (ML6), the crystal structure transitions into hexagonal closed packed (hcp) as the dominant phase. A figure highlighting this transition can be found in the Supporting Information (see Figure S2). This change in crystal structure can explain why the Ni‐Ru library in particular could not be predicted accurately, as the model is tasked with learning the activity distribution while the crystal structure remains unaccounted for, thereby reducing its predictive accuracy. Ensuring that the experiments are conducted in single‐phase compositional regions could help to reduce the complexity and the influence of the crystal structure on model performance in future studies. Similarly, incorporating high‐throughput electrochemical surface area (ECSA) measurements^[^ ^23^ ^]^ could address the influence of morphology by weighting the activity appropriately.

Apart from missing features, the twelve libraries inside the training dataset collectively cover only ≈36% of the quaternary composition space, providing insufficient data for accurate predictions. The sparse data coverage limits the model's ability to generalize, particularly in unexplored regions. Libraries farther away from the majority of observations, such as the binary Ni‐Ru (ML6) or the Pd‐Ru‐rich library (ML7) show a much greater prediction error when excluded from the training data compared to libraries in more densely sampled compositional regions, such as the equiatomic (ML1) or Pt‐rich libraries (ML4). As a non‐parametric algorithm, a Gaussian process only makes very few assumptions about the underlying function, e.g. via the chosen kernel function. While this flexibility makes it versatile, it also weakens its predictive performance in regions with sparse or no training data. This was addressed in this study by initializing the Bayesian optimization with five materials libraries covering a broader compositional space instead of a single one, making the initial predictions more robust.

Beyond these considerations, the approach of using current density values at a fixed potential to represent catalytic activity may not adequately capture the complexity of the system. While this metric is computationally simple and intuitive, it assumes linear relations within the chosen potential region. However, newly observed LSVs may deviate from this assumption, showing nonlinear behavior or potential shifts. For instance, pure Ru shows a plateau in current density beyond 1.6 V versus RHE, leading the model to underestimate its activity since it cannot account for other features of the LSV.

**Figure 9:**
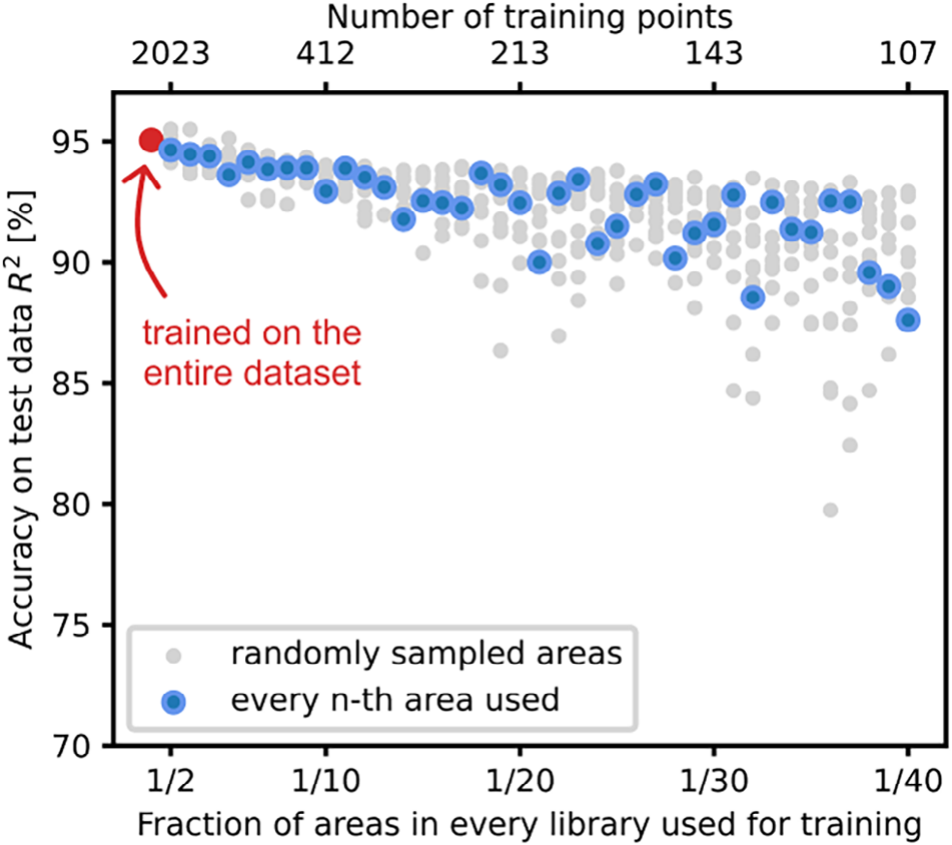
Effect of reducing the fraction of training data on the model's predictive accuracy (quantified by the coefficient of determination R^2^). Blue data points denote the accuracy when trained on every n^th^ measurement area of each library, while grey points indicate the accuracy when trained on randomly sampled areas. The model achieves an R^2^ of 95% when trained on the full dataset, demonstrating its ability to generalize the training data. Even when trained on as little as 1/20^th^ of the available data, the model maintains an R^2^ exceeding 90%, indicating a modest decrease in accuracy. The equivalent dataset size is shown on the secondary axis. The size depends on the number of areas excluded manually due to SDC measurement errors.

While the model struggles with accurately predicting entire materials libraries left out of the training dataset, its performance remains relatively consistent when data points are removed from each library. By excluding every second, third, and higher fraction of data points, the model still achieves a coefficient of determination (R^2^) exceeding 90%, even when trained on 1/20^th^ of the available data. This is visualized in **Figure** **9**, which shows the R^2^ values as a function of the fraction of data points used for training. When the model is trained on the full dataset, it achieves an R^2^ of 95%, demonstrating its ability to generalize the training data.

When fractions of the dataset are used for training, the accuracy decreases modestly as the fraction becomes smaller. For smaller fractions, the variability of R^2^ increases, as the selection of specific data points becomes more critical with reduced dataset size. Consequently, the reliability of the shown relationship decreases for smaller fractions. This is highlighted by grey data points, showing the accuracy when the selection of measurement areas was performed randomly. However, the figure suggests that measuring up to one‐fifth of the 342 pre‐defined areas on each library results is a tolerable decrease in accuracy while significantly improving experimental efficiency. The slight decrease in accuracy when leaving out data points can be attributed to the fact that the compositions and activities within each library vary only by small magnitudes relative to the size of the overall composition space and the distances between different libraries. This suggests that the fabrication of more libraries should be favored over the characterization of all measurement areas in a library. This is expected to become even more significant when exploring quinary composition spaces, as the local compositional gradients within a single library are even smaller relative to the overall composition space.

**Figure 10:**
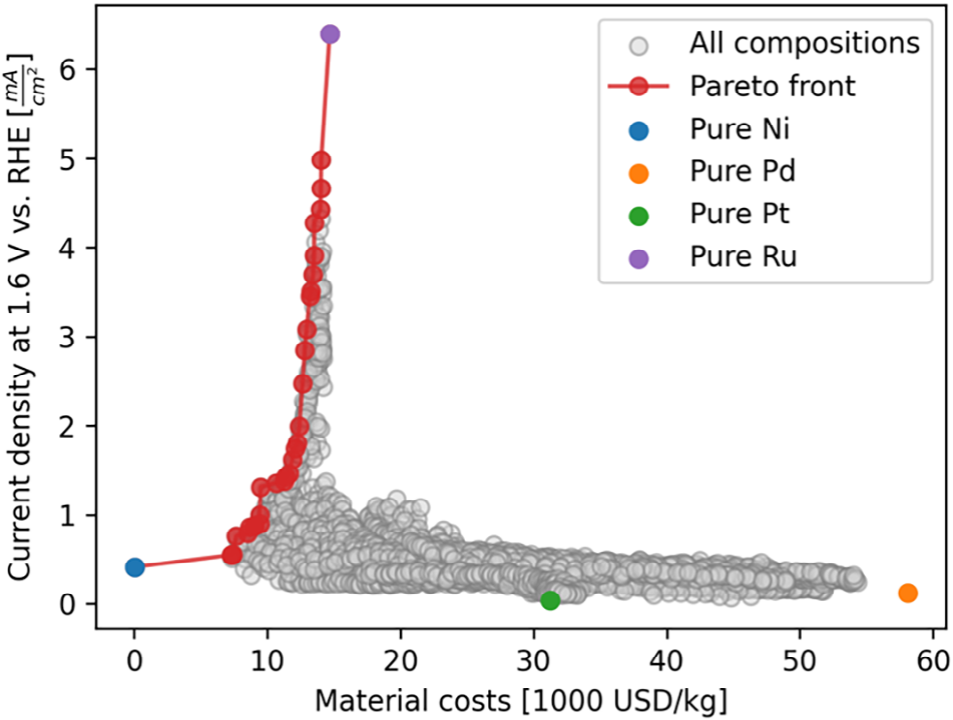
Trade‐off between the activity of the fabricated compositions and the material costs. The costs were calculated by weighing the raw material prices by the volume composition. The current density at 1.6 V versus RHE was chosen as an activity measure in order to avoid the plateau of the LSVs in the Ru‐rich areas likely caused due to mass transport limitations and bubble formation during the measurement. The Pareto front spans between pure Ru and pure Ni and highlights the steep increase in activity with higher Ru‐content on ML6.

To assess the trade‐off between catalytic activity and material cost, a Pareto front analysis was performed, taking into account the average raw material prices of the four elements from 2020–2024.^[^ ^24^ ^]^ **Figure** **10** shows the resulting trade‐off, with the Pareto front spanning from pure Ni to pure Ru. Most of the Pareto front is dominated by compositions from the binary Ni‐Ru library (ML6), which forms the steep vertical segment of the front. This indicates that while Ni partially improves activity compared to Pd or Pt, the highest activities are only reached at high Ru content. Pd and Pt compositions lie far from the Pareto front, confirming their limited contribution to activity in this system. The steep incline reflects decreasing gains in cost efficiency as Ru is substituted with Ni, suggesting that reducing Ru content leads to a rapid loss in activity.

## 4 Conclusion

Bayesian optimization can guide researchers efficiently through high‐dimensional search spaces. In the case of the explored Ni‐Pd‐Pt‐Ru system, the approach enabled the efficient identification of the global activity maximum after the fabrication and characterization of only six materials libraries, covering ≈20% of the quaternary composition space. Pure Ru was confirmed to be the most active OER catalyst in this system. Six additional libraries were fabricated, and their characterization validated the activity trend.

Despite the experimental effort of investigating more than 4000 compositions, the analysis showed that achieving comprehensive coverage of a quaternary composition space is experimentally costly, even when using combinatorial synthesis techniques and high‐throughput characterization. The explored regions correspond to ≈37% of the quaternary composition space, highlighting the challenge of achieving complete coverage. Due to this sparse data coverage, the employed Gaussian process model showed limited performance when extrapolating to unexplored regions. Libraries located farther away from the training data exhibited higher prediction errors. Nonetheless, the model showed high robustness when interpolating within observed regions of the composition space. Iteratively removing points from the training data showed that even when trained on 1/20^th^ of the available data, the model maintained a high predictive accuracy of more than 90%. This suggests that with the advantage of co‐sputtering compositional gradients, the exploration efficiency can be significantly improved compared to one‐at‐a‐time experiments. However, since the entire compositional gradient is not needed for accurate predictions, the number of measurements on a materials library should be reduced for future composition space explorations. In turn, the fabrication of more libraries should be preferred in order to sample more regions of the composition space. While this study demonstrates the feasibility of combining combinatorial synthesis directed by a combination of human decisions and Bayesian optimization for catalyst discovery, future work could benefit from benchmarking the approach against alternative sampling strategies to further quantify its efficiency.

This work demonstrates the potential of combining combinatorial synthesis with Bayesian optimization and expert guidance for the efficient exploration of multinary composition spaces and the reduction of experimental costs, providing a foundation for extending this methodology to even more complex materials systems. This is important for the goal of understanding and finding optimal electrocatalysts for industrial applications in the hydrogen economy.

## Conflict of Interest

The authors declare no conflict of interest.

## Author Contributions

F. Thelen and R. Zehl contributed equally to this work. F. Thelen performed conceptualization, sample fabrication, measurement, data curation, formal analysis, investigation, software, validation, visualization, supervision, wrote, reviewed, and edited the final manuscript. R. Zehl performed conceptualization, sample fabrication, measurement, investigation, supervision, wrote, reviewed, and edited the final manuscript. R. Zehl performed measurement, wrote the final manuscript. J. L. Bürgel performed sample fabrication, measurement, wrote, reviewed, and edited the final manuscript. L. Banko performed conceptualization, wrote, reviewed, and edited the final manuscript. W. Schuhmann performed supervision, resources, wrote, reviewed, and edited the final manuscript. A. Ludwig supervision, resources, project administration, wrote, reviewed, and edited the final manuscript.

## Supporting information



Supporting Information

## Data Availability

The data that support the findings of this study are openly available in ZENODO at https://doi.org/10.5281/zenodo.14891703, reference number 14891703.
