# Supplementary material for: Accelerating Combinatorial Electrocatalyst Discovery with Bayesian Optimization: a Case Study in the Quaternary System Ni‐Pd‐Pt‐Ru for the Oxygen Evolution Reaction
Source: Adv Sci (Weinh). 2025 Jun 29;12(35):e07302. doi: 10.1002/advs.202507302 (PMC12462997; doi:10.1002/advs.202507302)
Supplement: Supplementary file 1 — Supporting Information [file ADVS-12-e07302-s001.pdf]

ADVANCED SCIENCE
Open Access

Supporting Information

for *Adv. Sci.*, DOI 10.1002/advs.202507302

Accelerating Combinatorial Electrocatalyst Discovery with Bayesian Optimization: a Case Study in the Quaternary System Ni-Pd-Pt-Ru for the Oxygen Evolution Reaction

*Felix Thelen, Rico Zehl, Ridha Zerdoumi, Jan Lukas Bürgel, Lars Banko, Wolfgang Schuhmann and Alfred Ludwig**

Accelerating Combinatorial Electrocatalyst Discovery with Bayesian Optimization: A Case Study in the Quaternary System Ni-Pd-Pt-Ru for the Oxygen Evolution Reaction

Supporting Information

F. Thelen[1], R. Zehl[1], R. Zerdoumi[1,2], J. L. Bürgel[1], L. Banko[1], W. Schuhmann[2], A. Ludwig[1,*]

[1]Chair for Materials Discovery and Interfaces, Institute for Materials, Faculty of Mechanical Engineering, Ruhr University Bochum, Universitätsstraße 150, 44801 Bochum, Germany

[2]Analytical Chemistry– Center for Electrochemical Sciences (CES), Faculty of Chemistry and Biochemistry, Ruhr University Bochum, Universitätsstraße 150, 44801 Bochum, Germany

Underlined authors contributed equally.

*corresponding author: alfred.ludwig@rub.de

Contents:

Experimental section 2

Bayesian optimization 3

Coverage determination 3

Analysis of the crystal structure 4

Pareto front analysis 5

References 5

Dataset figures 7

Experimental section

Twelve materials libraries in the system Ni-Pd-Pt-Ru were fabricated at room temperature in a four-cathode co-sputter system (AJA International Polaris). The depositions were performed on 10 cm diameter single-side polished sapphire wafers (SITUS Technicals, c-plane orientation). Three DC power supplies (2x DCXS-750, 1x DCXS-1500) and a 0313 GTC RF unit (all AJA International) were used in power-control mode. The gas flow rate was 80 sccm Ar at a pressure of 0.5 Pa. An overview of the sputter parameters can be found in Table S-1. The deposition powers were determined based on preliminary sputter rate determinations and Monte Carlo sputter deposition simulations using pySIMTRA [1]. Due to the high sputter yield of Pd, the cathode equipped with Pd was assigned to an RF power supply. The single element thin films were deposited under identical conditions with a power of 35 W.

Table S-1: Power setpoint values used for each deposition and cathode.

		Ni	Pd	Pt	Ru
		Power [W]			
Library		DC	RF	DC	DC
ML1	Equiatomic	32	32	15	30
ML2	Ni-rich	45	11	6	8
ML3	Ru-rich	5	7	5	35
ML4	Pt-rich	9	7	35	5
ML5	Pd-rich	5	60	5	5
ML6	Ni-Ru	15	-	-	40
ML7	Pd-Ru-rich	9	80	5	80
ML8	Pd-Pt-Ru	-	40	19	34
ML9	Ni-Pt-Ru (high Ru)	42	-	8	60
ML10	Ni-Pt-Ru (high Pt)	45	-	55	7
ML11	Ni-Pd-Ru	60	100	-	7
ML12	Ni-Pd-Pt	52	14	30	-

The chemical composition was determined by automatically measuring 342 areas on each library using energy-dispersive X-ray spectroscopy (EDX) in a scanning electron microscope (SEM, JEOL L 7200F) equipped with an EDS detector (Oxford AZtecEnergy X-MaxN 80 mm^2). The electrochemical activity was measured with an automated scanning droplet cell (SDC) setup in a conventional three-electrode configuration, capable of automatically approaching all 342 areas as well. A Pt wire served as a counter electrode and a Ag|AgCl|3M KCl electrode as a reference electrode. The electrochemical cell is formed upon pressing the SDC tip onto the library, creating a circular contact area of 0.00735 cm^2, defined by the 1 mm tip opening. The applied force is monitored using a force sensor integrated into the tip holder. The OER activity was measured using linear sweep voltammetry in the potential range of 1.0 to 1.8 V vs. RHE at a scan rate of 10 mV s^{-1}. The measured current was normalized to the geometric surface area of the tip and no iR drop compensation was performed. All potentials were calculated and reported versus RHE using the following equation, where $E_{(Ag|AgCl|3M\ KCl)}$ is the measured potential versus Ag|AgCl|3M KCl:

$$E_{RHE} = E_{(Ag|AgCl|3M\ KCl)} + 0.210 + 0.059\,pH$$

In order to obtain a one-dimensional activity measure from the LSVs the current density at a potential of 1.7 V (vs. RHE) was extracted. Outlier detection of the LSVs was performed manually.

Bayesian optimization

For guiding the materials library synthesis experiments through the Ni-Pd-Pt-Ru composition space, a Gaussian process implemented in GPflow [2] was used in a Bayesian optimization loop in order to find the composition with the highest catalytic activity for the OER. The Gaussian process was supplied with the measured compositions (values between 0 and 1) as input and the activity measure as output training data. The training data is supplied to the model "as is", e.g. without standardization. Since the shape of the multi-dimensional function, which should be learned by the Gaussian process, is unknown in advance, the Matérn52 kernel was selected. It can provide a more flexible fit due to a higher number of hyperparameters compared to the most commonly used squared exponential kernel [3,4]. Also, it was found to be more performant on a similar study on co-sputtered materials libraries [5]. The predictions were done on the entire composition space sampled at 5 at.% steps. The compositions to fabricate next were determined by maximizing the expected improvement acquisition function based on the predicted activity and the uncertainty of the Gaussian process. The expected improvement EI is defined as

$$EI(x) = (\mu(x) - f_{max}) \cdot \Phi\left(\frac{\mu(x) - f_{max}}{\sigma(x)}\right) + \sigma(x) \cdot \phi\left(\frac{\mu(x) - f_{max}}{\sigma(x)}\right)$$

where $\mu(x)$ denotes the predicted mean at point x and $\sigma(x)$ the predicted standard deviation from the Gaussian process, f_{max} is the highest observed value so far and Φ and ϕ denote the cumulative distribution function as well as the probability density function of the standard normal distribution respectively [6,7].

Coverage determination

To quantify the exploration progress in covering the composition space of interest, a coverage metric was defined, which specifies the amount of an n-dimensional composition space covered by a set of p-compositions $C_f = \{c_{f,i}\}_{i=1}^{p}$. A second set of compositions $C_s = \{c_{s,i}\}_{i=1}^{q}$ is obtained by sampling q-compositions from the composition space with a step size of $t\ at.\%$ according to

$$q = \binom{T - n - 1}{n - 1}$$

with $T = 100 \cdot t^{-1}$ being the number of partitions along each composition space axis. This is shown in Figure S-1 for a ternary materials library, which covers 30.3% of the ternary composition space.

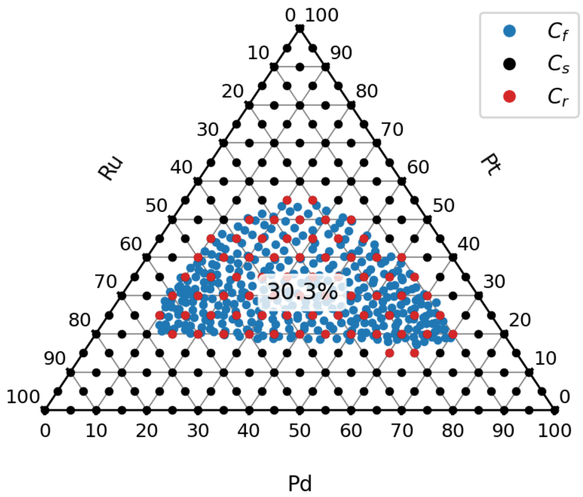

Figure S-1: Plot visualizing the coverage calculation. The compositions of a materials library obtained by EDX are shown in blue. All possible compositions in 5 at. % steps are shown in black and all nearest neighbors to the measured compositions in red. The coverage is defined as the ratio of the number of points in C_r and the total number of combinations in C_r, which corresponds to 70/231.

For a quaternary composition space sampled with steps of $5\,at.\%$, this corresponds to $q = 1771$ compositions. When there is a set of r-nearest neighbors of C_s in C_f, called C_r, the coverage cov of the compositions C_f is defined as the ratio of the number of nearest neighbors r to the total number of sampled compositions q:

$$cov = \frac{r}{q} \cdot 100\ [\%]$$

Analysis of the crystal structure

To illustrate the change in crystal structure across the composition space, the hcp (101) peak was tracked in the two-theta range of 42° - 46°, as shown in Figure S-2b. This peak was observed exclusively in libraries ML3, ML6, ML7 and ML9, with the highest intensity detected in ML6. Figure S-2a shows exemplary diffractograms highlighting this trend. Towards higher Ru content, the hcp peaks become increasingly visible, indicating a structural transition. In contrast, throughout the remainder of the composition space, only an fcc phase was detected. The observed trend suggests that Ni-Ru combinations favor the formation of the hcp phase, whereas mixtures with other elements exhibit the hcp structure only at high Ru concentrations.

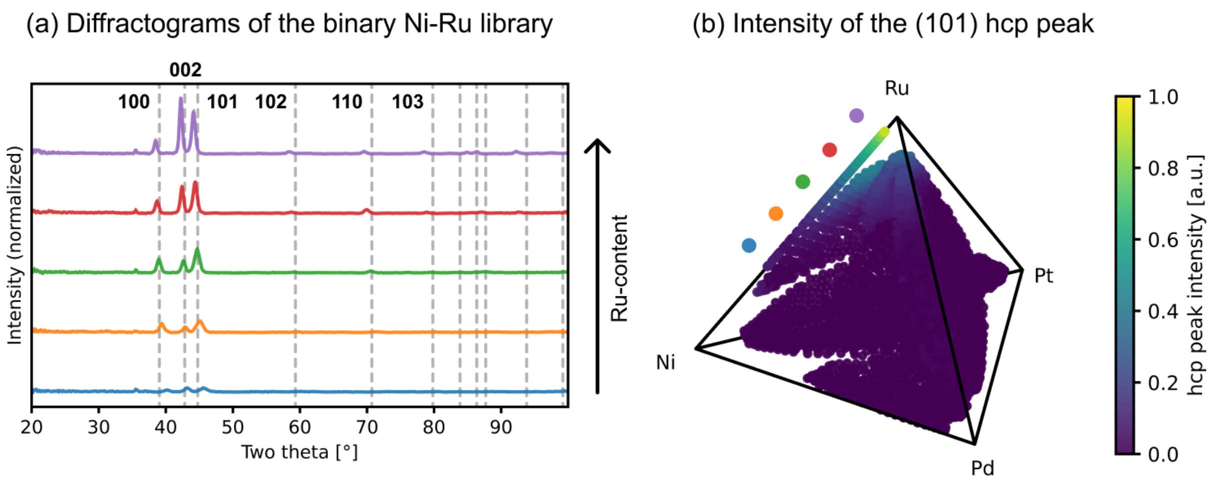

Figure S-2: (a) Exemplary XRD diffractograms of the binary Ni-Ru library along the compositional gradient. (b) Intensity map of the hcp (101) peak in the composition space. The peak is only visible in the libraries ML3, ML6, ML7 and ML9. The intensity of this peak increases with higher Ru content, with the strongest signal observed in ML6, while the rest of the composition space shows only an fcc phase.

Pareto front analysis

In order to assess the trade-off between activity and material costs, the raw material prices of Ni, Pd, Pt and Ru were weighted by the volume composition of each material and summed consecutively. The average prices for 2020-2024 were taken from the latest report of the German Raw Materials Agency [8] (March 2025).

Table S-2: Material costs of the four elements.

Element	Ni	Pd	Pt	Ru
Price in USD/kg	19.27	58,074.85	31,247.75	14,675,28

References

[1] F. Thelen, R. Zehl, J.L. Bürgel, D. Depla, A. Ludwig, A python-based approach to sputter deposition simulations in combinatorial materials science, Surf Coat Technol 503 (2025) 131998. https://doi.org/10.1016/j.surfcoat.2025.131998.

[2] A.G. de G. Matthews, M. van der Wilk, T. Nickson, K. Fujii, A. Boukouvalas, P. Leon-Villagra, Z. Ghahramani, J. Hensman, GPflow: A Gaussian process library using TensorFlow, Journal of Machine Learning Research 18 (2017) 1–6. http://jmlr.org/papers/v18/16-537.html (accessed May 24, 2023).

[3] C.E. Rasmussen, C.K.I. Williams, Gaussian Processes for Machine Learning, The MIT Press, Massachusetts, 2006.

[4] B. Matérn, Spatial variation, 2nd ed., Springer Verlag Berlin-Heidelberg GmbH, Enebyberg, 2013. https://doi.org/10.1007/978-1-4615-7892-5.

[5] F. Thelen, L. Banko, R. Zehl, S. Baha, A. Ludwig, Speeding up high-throughput characterization of materials libraries by active learning: autonomous electrical resistance measurements, Digital Discovery 2 (2023) 1612–1619. https://doi.org/10.1039/D3DD00125C.

[6] D.R. Jones, M. Schonlau, W.J. Welch, Efficient Global Optimization of Expensive Black-Box Functions, Journal of Global Optimization 13 (1998) 455–492. https://doi.org/10.1023/A:1008306431147.

[7] E. Brochu, V.M. Cora, N. De Freitas, A Tutorial on Bayesian Optimization of Expensive Cost Functions, with Application to Active User Modeling and Hierarchical Reinforcement Learning, ArXiv Preprint (2010). https://doi.org/10.48550/arXiv.1012.2599.

[8] D. Bastian, L. Prochaska, M. Kern, Rohstoff Preismonitor, Berlin, 2025. https://www.deutsche-rohstoffagentur.de/DERA/DE/Produkte/Rohstoffpreise/Preismonitor/preismonitor_node.html (accessed April 17, 2025).

Dataset figures

Figures S-3-14: LSVs and activity gradients of all 12 materials libraries.

ML1: equiatomic library

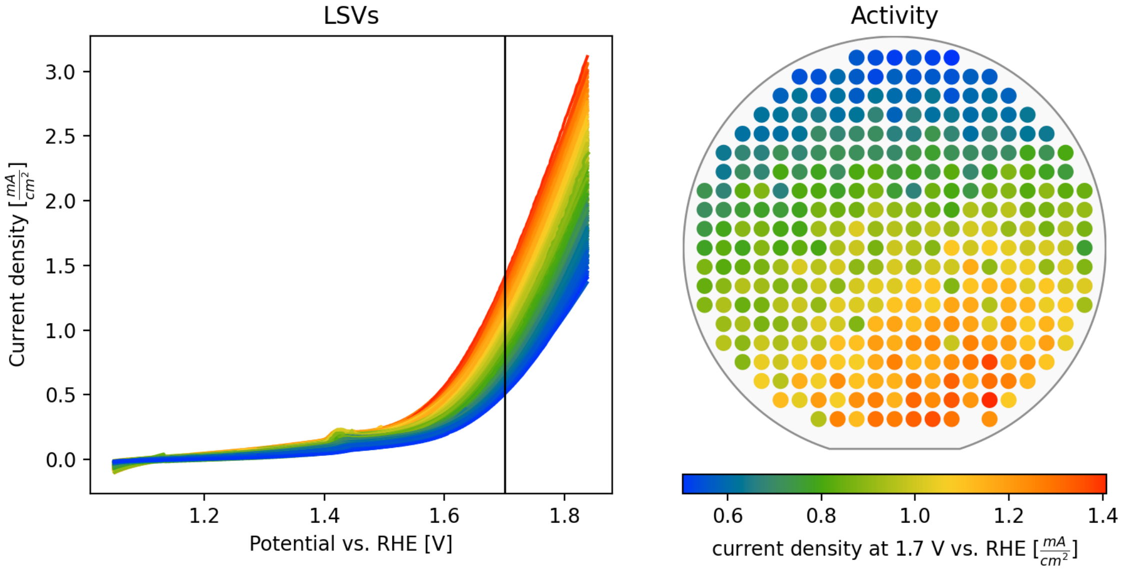

ML2: Ni-rich library

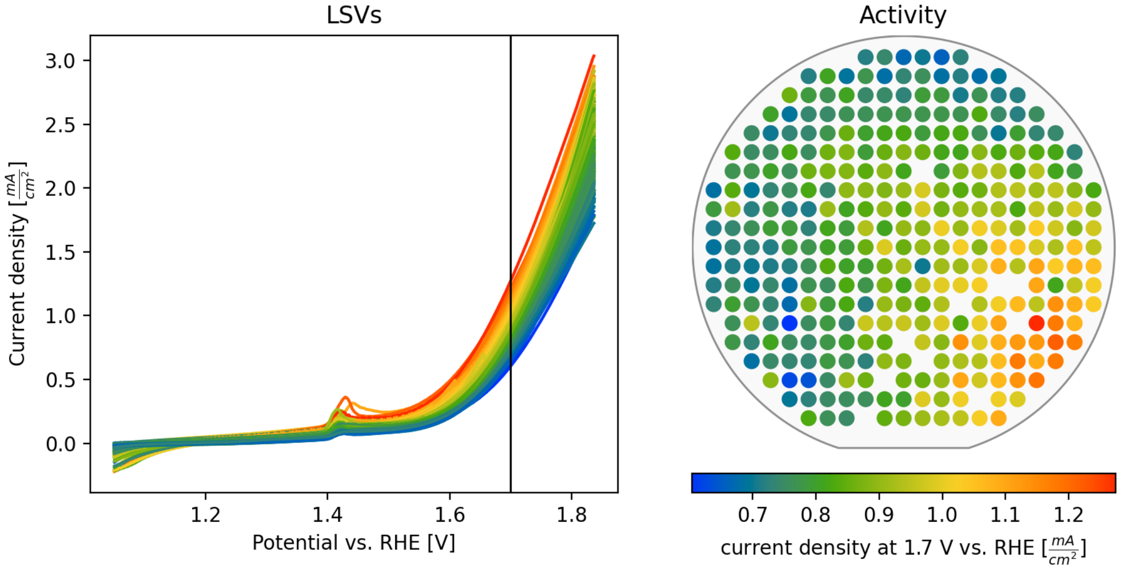

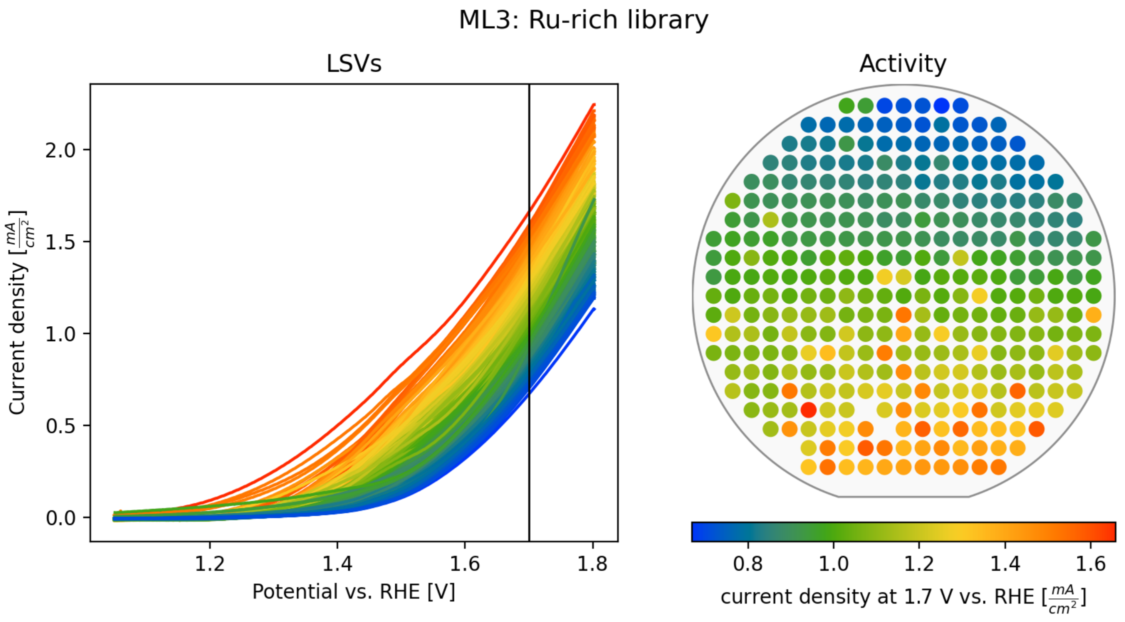
ML3: Ru-rich library
LSVs
Activity
Current density [$\frac{mA}{cm^2}$]
Potential vs. RHE [V]
current density at 1.7 V vs. RHE [$\frac{mA}{cm^2}$]

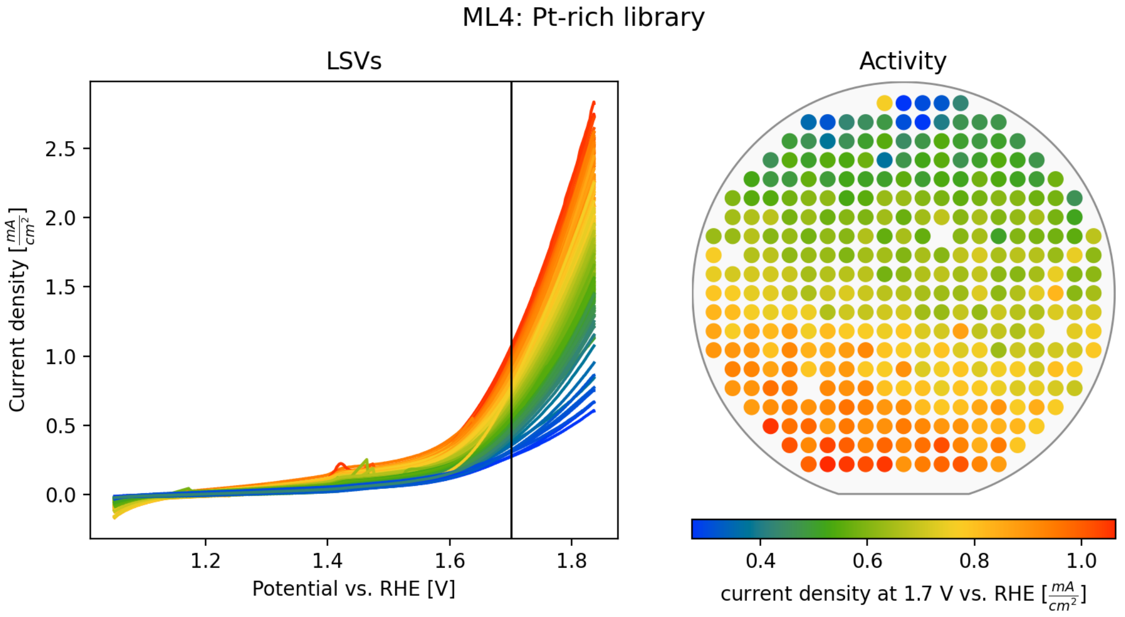
ML4: Pt-rich library
LSVs
Activity
Current density [$\frac{mA}{cm^2}$]
Potential vs. RHE [V]
current density at 1.7 V vs. RHE [$\frac{mA}{cm^2}$]

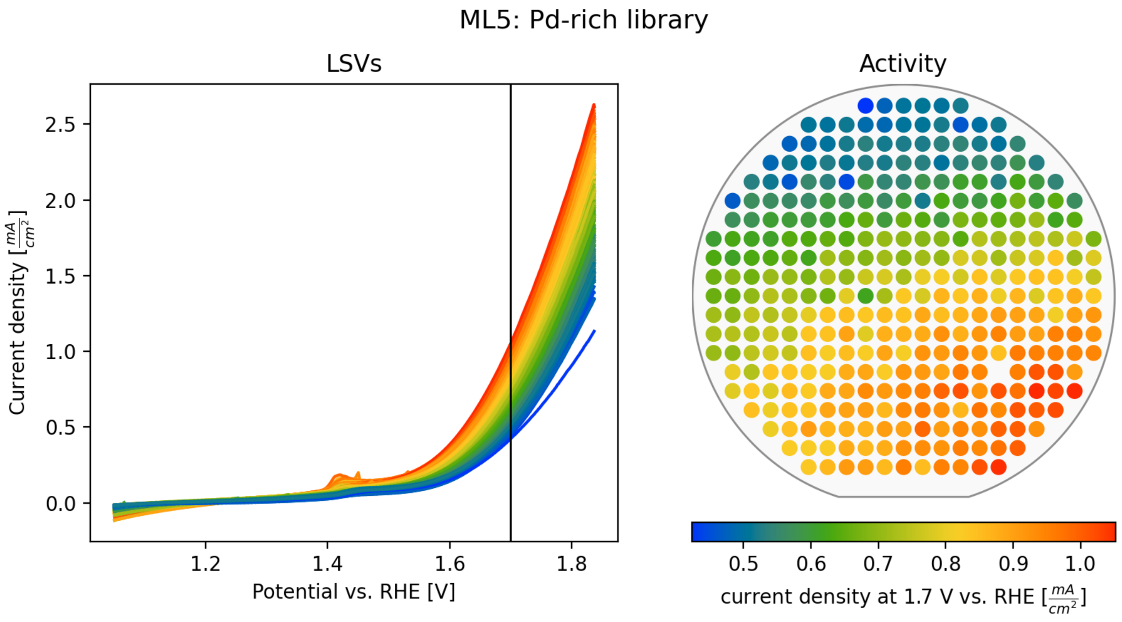
ML5: Pd-rich library
LSVs
Activity
Current density [mA/cm²]
Potential vs. RHE [V]
current density at 1.7 V vs. RHE [mA/cm²]
2.5
2.0
1.5
1.0
0.5
0.0
1.2
1.4
1.6
1.8
0.5
0.6
0.7
0.8
0.9
1.0

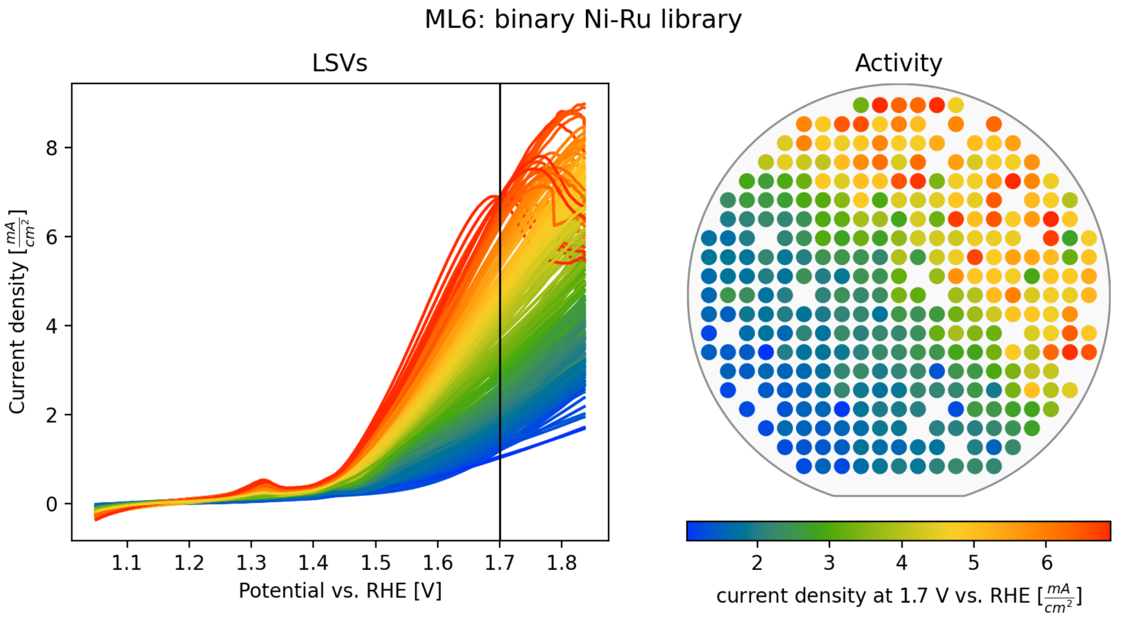
ML6: binary Ni-Ru library
LSVs
Activity
Current density [mA/cm²]
Potential vs. RHE [V]
current density at 1.7 V vs. RHE [mA/cm²]
8
6
4
2
0
1.1
1.2
1.3
1.4
1.5
1.6
1.7
1.8
2
3
4
5
6

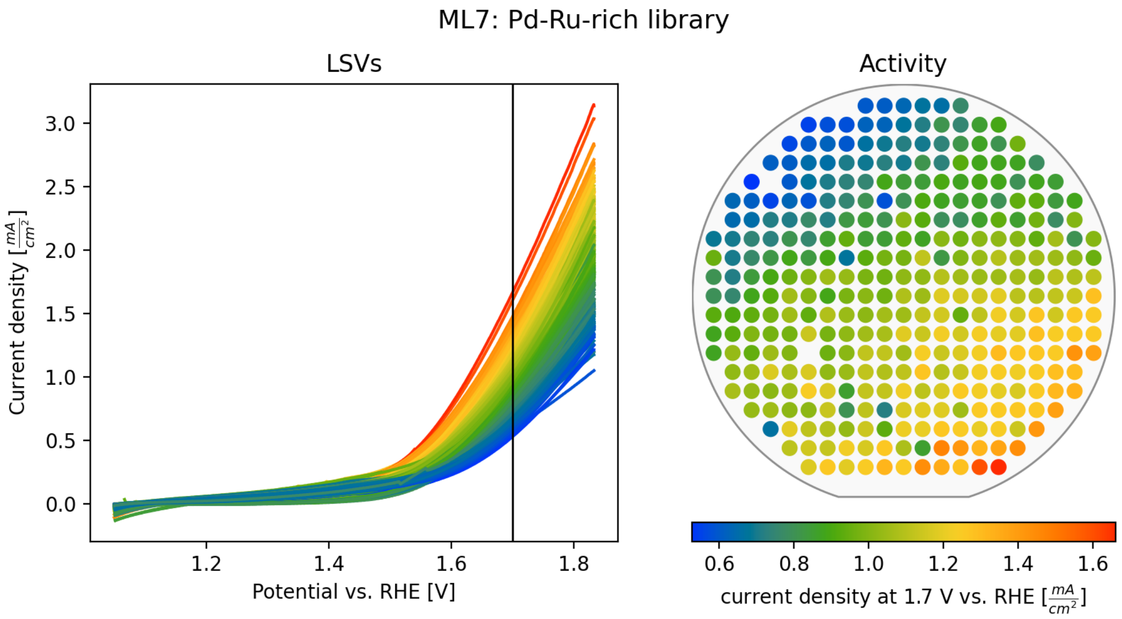
ML7: Pd-Ru-rich library
LSVs
Activity
Current density [mA/cm²]
0.0
0.5
1.0
1.5
2.0
2.5
3.0
1.2
1.4
1.6
1.8
Potential vs. RHE [V]
0.6
0.8
1.0
1.2
1.4
1.6
current density at 1.7 V vs. RHE [mA/cm²]

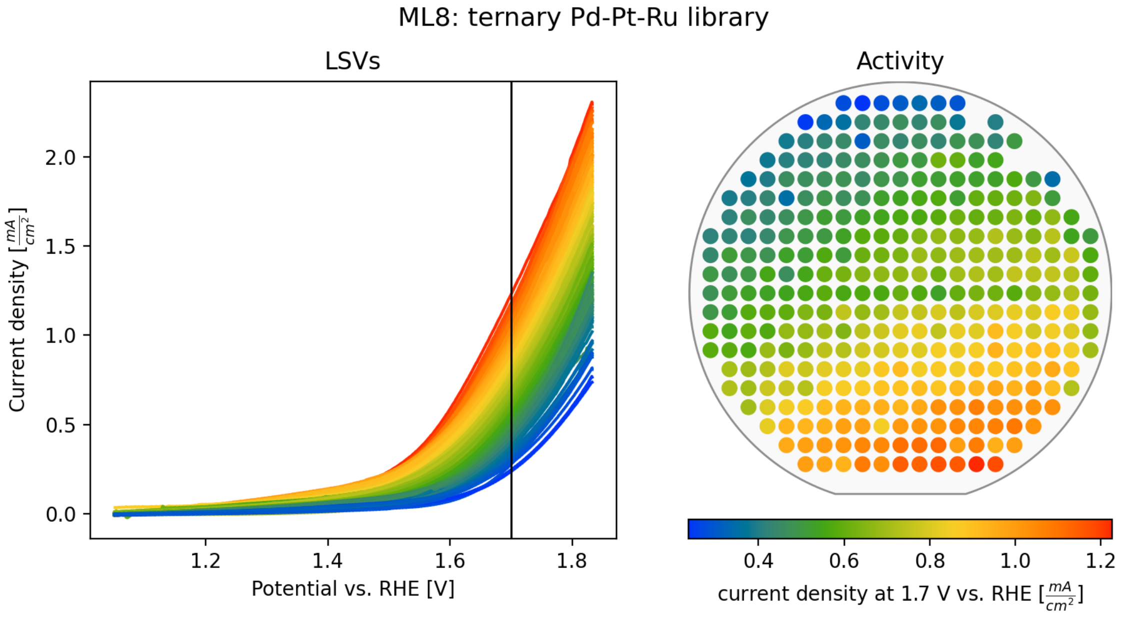
ML8: ternary Pd-Pt-Ru library
LSVs
Activity
Current density [mA/cm²]
0.0
0.5
1.0
1.5
2.0
1.2
1.4
1.6
1.8
Potential vs. RHE [V]
0.4
0.6
0.8
1.0
1.2
current density at 1.7 V vs. RHE [mA/cm²]

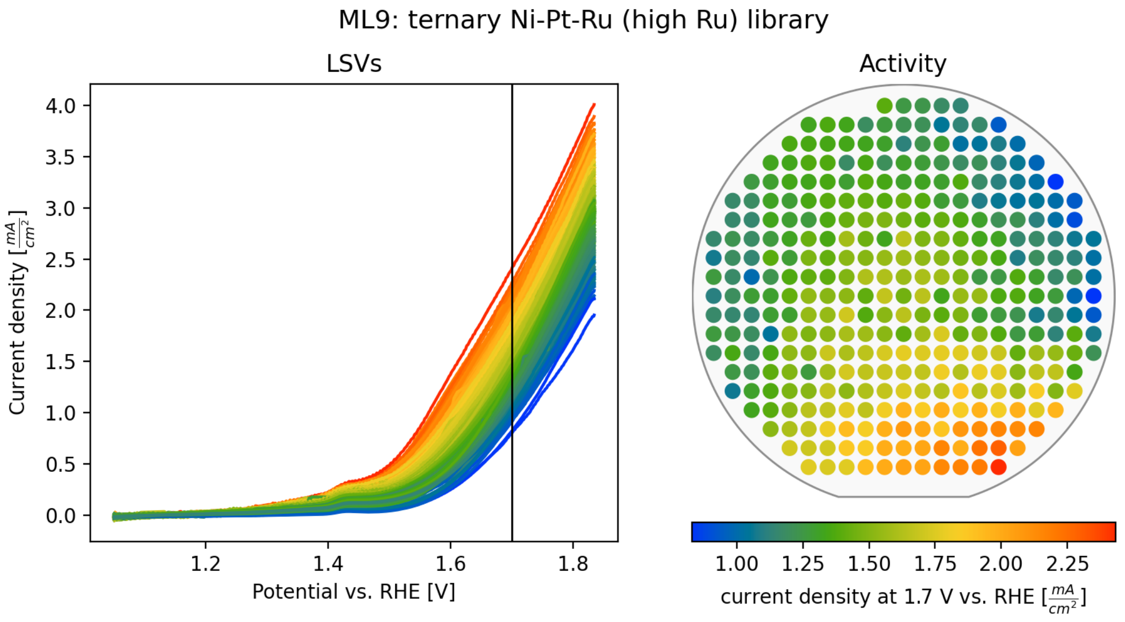
ML9: ternary Ni-Pt-Ru (high Ru) library
LSVs
Activity
Current density [$\frac{mA}{cm^2}$]
Potential vs. RHE [V]
current density at 1.7 V vs. RHE [$\frac{mA}{cm^2}$]

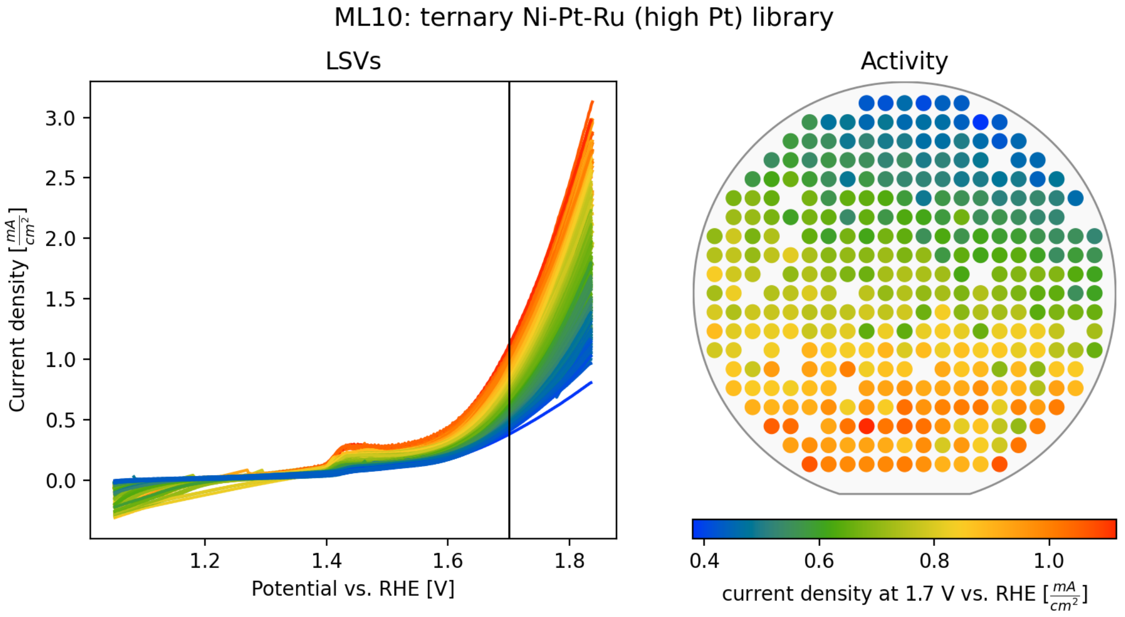
ML10: ternary Ni-Pt-Ru (high Pt) library
LSVs
Activity
Current density [$\frac{mA}{cm^2}$]
Potential vs. RHE [V]
current density at 1.7 V vs. RHE [$\frac{mA}{cm^2}$]

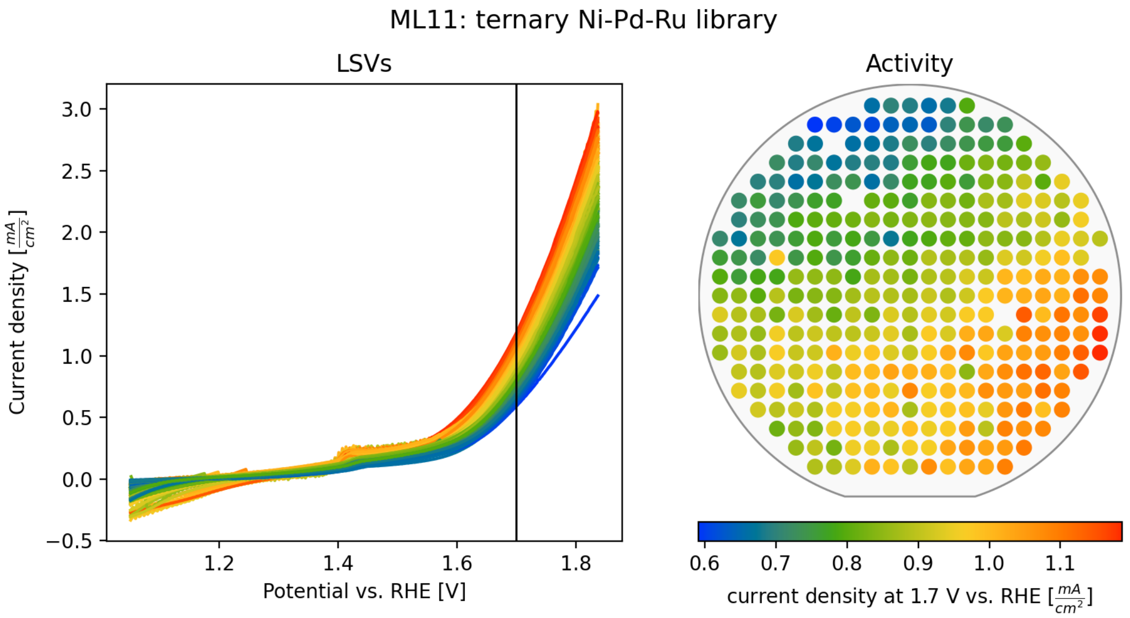
ML11: ternary Ni-Pd-Ru library
LSVs
Activity
Current density [$\frac{mA}{cm^2}$]
Potential vs. RHE [V]
current density at 1.7 V vs. RHE [$\frac{mA}{cm^2}$]

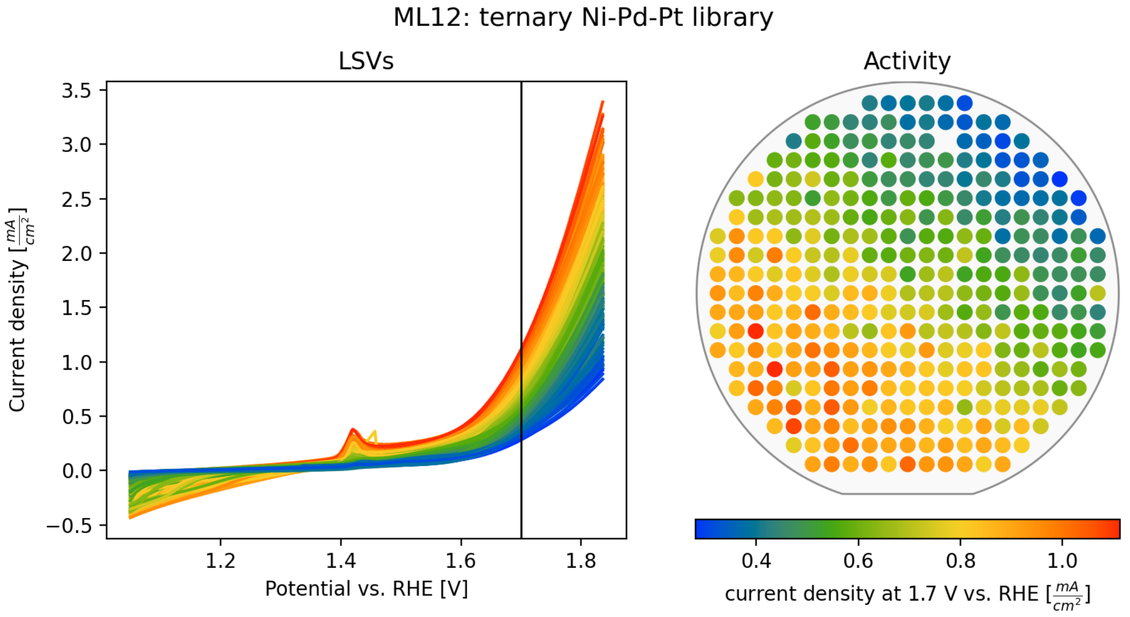
ML12: ternary Ni-Pd-Pt library
LSVs
Activity
Current density [$\frac{mA}{cm^2}$]
Potential vs. RHE [V]
current density at 1.7 V vs. RHE [$\frac{mA}{cm^2}$]
